# The influence of serum, glucose and oxygen on intervertebral disc cell growth *in vitro*: implications for degenerative disc disease

**DOI:** 10.1186/ar2405

**Published:** 2008-04-23

**Authors:** William EB Johnson, Simon Stephan, Sally Roberts

**Affiliations:** 1 Centre for Spinal Studies, Robert Jones and Agnes Hunt Orthopaedic Hospital, Oswestry, Shropshire, SY10 7AG, UK; 2 Institute for Science and Technology in Medicine, Keele University, Keele, Staffordshire, ST5 5BG, UK

## Abstract

**Introduction:**

The avascular nature of the human intervertebral disc (IVD) is thought to play a major role in disc pathophysiology by limiting nutrient supply to resident IVD cells. In the human IVD, the central IVD cells at maturity are normally chondrocytic in phenotype. However, abnormal cell phenotypes have been associated with degenerative disc diseases, including cell proliferation and cluster formation, cell death, stellate morphologies, and cell senescence. Therefore, we have examined the relative influence of possible blood-borne factors on the growth characteristics of IVD cells *in vitro*.

**Methods:**

Bovine IVD cells were cultured either in monolayer to encourage cell proliferation or in alginate to induce chondrocytic differentiation. In both culture systems, cells were maintained with or without 20% serum, with or without 320 mg/dL glucose, and in atmospheric levels (~21%) of oxygen or 1% oxygen. Cell proliferation and viability, cell senescence, and collagen immunopositivity were assessed after 7 days. Statistical differences in these growth characteristics were tested using nonparametric analyses (n = 4 samples).

**Results:**

In both culture systems, serum deprivation significantly inhibited IVD cell proliferation and increased cell positivity for senescence-associated beta-galactosidase (SA-β-gal), a marker of cell senescence. Conversely, IVD cells cultured in the presence of serum, but deprived of glucose, proliferated significantly more rapidly. In alginate cultures, this enhanced cell proliferation (through glucose deprivation) led to the formation of IVD cell clusters. Serum-deprived cells in monolayer, but not in alginate, adopted a stellate appearance. Oxygen deprivation alone had little effect on IVD cell proliferation or survival. Oxygen and glucose deprivation also had no significant effect on SA-β-gal positivity. IVD cell viability was markedly and significantly decreased in serum-deprived alginate cultures, but in all other conditions remained at or greater than approximately 95%. Glucose deprivation, but not serum or oxygen deprivation, inhibited synthesis of type I and type II collagen, both in monolayer and alginate cultures.

**Conclusion:**

This study demonstrates that factors present in serum interact with other nutrients, notably glucose, to play a major role in regulating the behaviour of IVD cells. These findings suggest that IVD cell phenotypes seen in degenerative disc disease may arise through the cells' response to altered vascularisation and nutrient supply.

## Introduction

The intervertebral disc (IVD) is the largest avascular connective tissue in the human body. It is composed largely of a collagenous extracellular matrix that is sparsely interspersed by IVD cells (~6,000 mm^-3^) [1]. The activity of IVD cells is important to tissue function, and IVD cell death, along with decreased IVD cellularity, is associated with ageing and degenerative disc pathology [2,3]. Abnormal cell behaviour seen in pathological human IVDs also includes increased cell proliferation and cluster formation [4], the appearance of stellate cells [5], and cell senescence [6-8]. The avascularity of the disc has long been considered to influence IVD cell function [1], a view supported by *in vitro *studies. Hence, Urban and colleagues [9] demonstrated that reduced levels of glucose and oxygen, combined with other environmental conditions present in the inner parts of human IVDs (that is, decreased pH and increased osmolarity), decrease the anabolic activity of IVD cells. Glucose deprivation, in particular, also results in increased IVD cell death, an effect that increases with increased cell density (see reviews [9,10]). There are also clinical and experimental indications that factors affecting vascular function or glucose supply are associated with an increased risk of disc disease. For example, atherosclerosis [11] and smoking [12], along with diabetes [13,14], have been linked to IVD degeneration, although the influence of insulin-dependent diabetes on susceptibility to human disc disease remains unproven [15].

Few studies have examined the influence of serum-derived factors on the growth and survival of IVD cells, even though these factors generally are considered to be essential for the survival of most normal cell types. This may be because workers in the field have considered it unlikely that growth factors enter the IVD's inner regions via diffusion from the peripheral vasculature. Indeed, one early study [16] demonstrated that the diffusion rate of molecules through the cartilage endplate, the major route of glucose and oxygen to the central nucleus pulposus (NP) [9,10], was dependent on their size, charge, and shape, with larger, more globular proteins diffusing more slowly than smaller, more linear molecules. However, there is no direct evidence that serum-derived factors are restricted from entering the IVD. Furthermore, the IVD becomes increasingly vascularised as it degenerates or is damaged, with blood vessels particularly entering disrupted regions of the tissue [17-19]. Herniated discs are also frequently vascularised [20,21]. Hence, the provision of serum-derived factors to IVD cells may increase in pathological IVDs and consequently play an important role in regulating IVD cell behaviour. In this study, we have examined the relative influence of serum, glucose, and oxygen supply, singly or combined, on the growth pattern of bovine IVD cells. Since the nutrient supply affects the NP more than the anulus fibrosus (AF), the response of NP disc cells was investigated.

## Materials and methods

### Intervertebral disc cell culture

IVD cells were isolated from the NP of adult bovine caudal IVDs by enzymatic digestion and expanded in monolayer culture in standard Dulbecco's modified Eagle's medium (DMEM)/F-12 supplemented with 10% foetal calf serum (FCS), penicillin, and streptomycin, as described [22] (all reagents from Invitrogen Ltd., Paisley, UK). Freshly isolated NP cells are chondrocytic in phenotype, but during monolayer culture, they become adherent and fibroblast-like in morphology and enter the cell cycle to proliferate, a process that has been termed chondrocyte dedifferentiation [23]. At passage II, these fibroblastic cells were seeded into culture plates at a density of 2 × 10^3 ^cells per square centimetre in DMEM containing 10% FCS and left to adhere overnight. Following washes with phosphate-buffered saline, seeded cells were fed with DMEM (a) supplemented with or without 20% FCS, (b) supplemented with or without 320 mg/dL glucose, and (c) maintained in a humidified atmosphere containing atmospheric levels (~21%) of oxygen or 1% oxygen. Various combinations of serum, glucose, and oxygen supplementation were also examined. Alternatively, passage II IVD cells were seeded into alginate beads at a low cell density of 10^6 ^cells per millilitre and a final alginate concentration of 1.2%. The alginate beads were then incubated in combinations of medium supplemented with or without serum and glucose and in 21% or 1% oxygen, as described above. All culture medium was at pH 6.9 to 7.1 and approximately 312 to 321 mOsm/kg water. Cultures were fed at day 4 after the initial feeding and harvested on day 7. For all analyses, control cultures were designated as those maintained at atmospheric levels of oxygen and in medium supplemented with 20% FCS and 320 mg/dL glucose.

### Cell proliferation and cell viability

Monolayers were harvested by trypsinisation and viable cell counts were performed using trypan blue. The proliferation status of cells was also examined by immunocytochemistry for the proliferation-associated Ki-67 antigen, as described [24]. Cell viability in alginate was assessed by 'live/dead' scoring, in which live cells fluoresce green and dead cells fluoresce red (Live/Dead; Invitrogen Ltd.) [25].

### Cell senescence

Senescent cells express much greater levels of beta-galactosidase activity than nonsenescent cells such that its enzymic activity can be detected at the suboptimal pH of 6.0 [26]. Hence, senescence-associated beta-galactosidase (SA-β-gal) activity was examined, as described [6], and the proportion of SA-β-gal-positive cells was quantitated by scoring a minimum of 200 cells for each condition and each sample.

### Collagen immunolocalisation

The production of type I and II collagens was examined by immunolocalisation of cytospins, as described [24]. In brief, formalin-fixed cytospins were incubated with antibodies specific for collagen type I (clone I-8H5; ICN Biomedical Ltd., now part of MP Biomedicals, Irvine, CA, USA) or collagen type II (clone C11C1; Developmental Studies Hybridoma Bank, Iowa City, IA, USA). Immunoreactivity was revealed using a commercial kit (Vectastain ABC Elite; Vecta Labs Ltd., Peterborough, UK) combined with streptavidin-FITC (fluorescein isothiocyanate) (Vecta Labs Ltd.) and counterstained with DAPI (4'-6-diamidino-2-phenylindole). Immunolocalisation was also performed using isotype-matched irrelevant antibodies (Dako, Ely, UK); this staining was negative. The presence of filamentous actin (F-actin) was assessed using fluorescently tagged phalloidin (FITC-phalloidin; Molecular Probes Inc., now part of Invitrogen Corporation, Carlsbad, CA, USA), as described [24].

### Microscopy and image capture

Cultures were viewed using phase-contrast and fluorescence microscopy (Nikon Eclipse TS100; Nikon, Kingston-upon-Thames, UK). Digitized images were captured with a Hamamatsu (C4742-95; Hamamatsu Corporation, Bridgewater, NJ, USA) or Nikon digital camera and examined using IP Lab software (version 3.6; Nikon).

### Statistical analysis

The nonparametric Mann-Whitney *U *test was used to assess significant differences between control cultures and cultures deprived of nutrient factors for the following parameters: (a) the harvested cell number, (b) the proportion of Ki-67-immunopositive cells, (c) cell viability, and (d) the proportion of SA-β-gal-positive cells. All data shown are presented as mean ± standard error of the mean, where NP cell cultures were established from caudal IVD obtained from four bovine tails (that is, n = 4). Significance differences were accepted at a *P *value of less than 0.05.

## Results

### Growth kinetics of monolayer cultures

**Figure 1:**
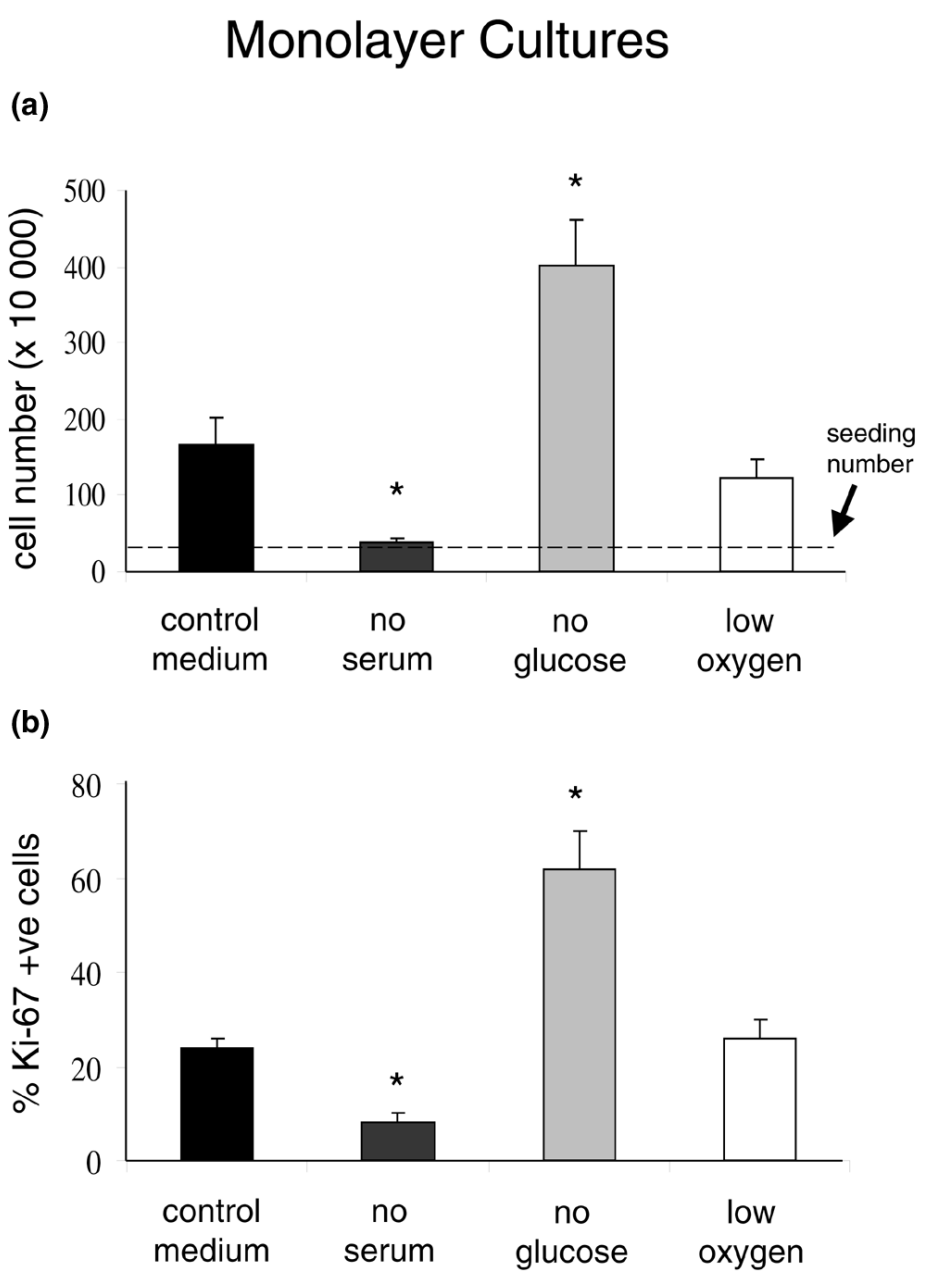
The growth of intervertebral disc cells in monolayer culture. **(a) **Compared with control cultures, cell numbers at day 7 were significantly decreased in serum-deprived cultures and increased in glucose-deprived cultures. Oxygen deprivation had no significant effect. **(b) **The proportions of Ki-67-immunopositive cells were also significantly decreased in serum-deprived cultures and increased in glucose-deprived cultures. Oxygen deprivation had no significant effect. Data are presented as mean ± standard error of the mean. *P < 0.05.

From a seeding population of 20 × 10^3 ^cells per well (in six-well plates), control monolayer cultures expanded to 166 ± 37 × 10^3 ^cells per well by day 7. In contrast, monolayer cultures in serum-free medium increased only to 37 ± 6 × 10^3 ^cells per well. Monolayers in glucose-deprived medium expanded to 403 ± 60 × 10^3 ^cells per well over the same time period (Figure 1a). These significant differences in cell harvests were reflected in the proportions of Ki-67-immunopositive cells (Figure 1b). The proliferative response of IVD cells to glucose deprivation was abrogated when cultures were further deprived either of serum or oxygen such that only 60 ± 9 × 10^3 ^cells per well or 198 ± 47 × 10^3 ^cells per well were harvested, respectively. Oxygen deprivation alone did not significantly alter the harvested cell number or the Ki-67 index. Day 7 cell harvests of serum-deprived cultures which were also further deprived of oxygen (31 ± 8 × 10^3 ^cells per well) or of oxygen and glucose (53 ± 21 × 10^3 ^cells per well) were not significantly different from those cultures deprived of serum alone. In all of these experiments, there was no evidence of monolayered cells becoming nonadherent and cell viability remained at 98% to 99%, except for glucose-deprived cultures in which viability by day 7 had decreased slightly, but significantly, to 94.8% ± 0.6% (Mann-Whitney *U *test; *P *= 0.0286). Hence, serum deprivation was associated with IVD cell growth arrest, whereas glucose deprivation (in the presence of serum and oxygen) resulted in IVD cell proliferation.

### Morphology and senescence

**Figure 2:**
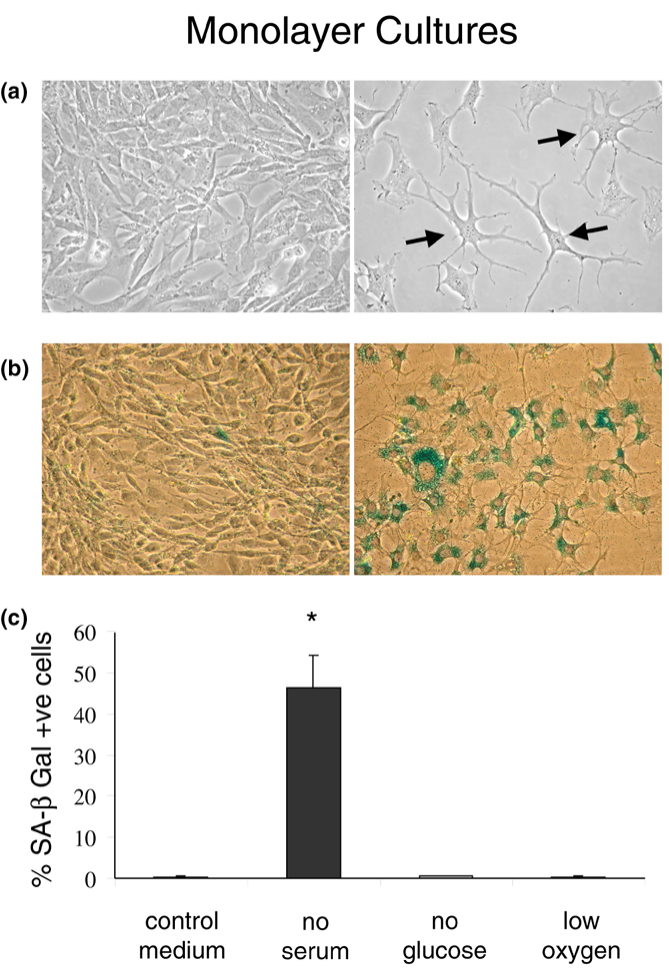
Serum deprivation of intervertebral disc cells in monolayer cultures was associated with the adoption of a stellate morphology and increased cell senescence. **(a) **Representative images of control (left panel) and serum-deprived (right panel) cells at day 7; stellate cells are indicated with arrows (original magnification ×200). **(b) **Representative images of control (left panel) and serum-deprived (right panel) cultures stained for senescence-associated beta-galactosidase (SA-β-gal) activity at day 7 (original magnification ×100). **(c) **The proportion of SA-β-gal-positive cells was significantly increased in serum-deprived cultures compared with control cultures but was unaffected by glucose or oxygen deprivation. Data are presented as mean ± standard error of the mean. *P < 0.05.

Serum-deprived IVD cells in monolayer, but not control or glucose- or oxygen-deprived cells, exhibited a stellate morphology, with many cells extending several branching cell processes (Figure 2a). Furthermore, the proportion of SA-β-gal-positive cells was significantly greater in serum-deprived cultures (46% ± 8%) compared with control or glucose- or oxygen-deprived cultures (~0.5%) (Figure 2b, c). However, there was no clear relationship between cell morphology and cell senescence. Increased SA-β-gal positivity was not seen in cultures deprived of glucose and/or oxygen, nor was it seen to any greater extent in serum-deprived cultures that were further deprived of glucose and/or oxygen (data not shown).

### Alginate cultures

**Figure 3:**
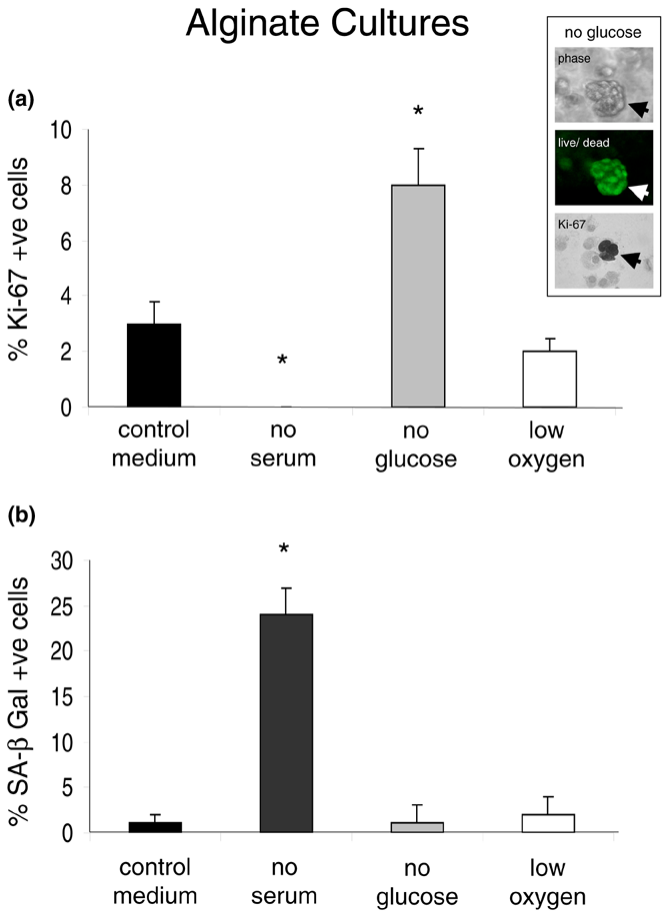
The growth and senescence of intervertebral disc (IVD) cells in alginate culture. **(a) **Compared with control cultures, the proportion of Ki-67-immunopositive cells was significantly decreased in serum-deprived cultures and increased in glucose-deprived cultures. Oxygen deprivation had no significant effect. Inset: representative images of glucose-deprived alginate cultures, showing a viable cell cluster, and Ki-67-immunopositive cells following harvest (arrow) (original magnification ×200). **(b) **The proportion of senescence-associated beta-galactosidase (SA-β-gal)-positive IVD cells was significantly increased in serum-deprived cultures compared with control cultures but was unaffected by glucose or oxygen deprivation. Data are presented as mean ± standard error of the mean (n = 4). *P < 0.05.

IVD cells cultured in alginate were markedly less proliferative than those in monolayer. Hence, whereas 24% ± 2% of cells in control monolayers were Ki-67-immunopositive, only 3% ± 0.8% of cells were positive in control alginate cultures. None of the cells in serum-deprived alginate cultures was Ki-67-immunopositive. The proportion of Ki-67-immunopositive cells was significantly greater in glucose-deprived alginate cultures (8% ± 1.3%) compared with controls, whereas oxygen deprivation had no significant effect (Figure 3a). Therefore, and similar to our observations in monolayer, serum deprivation of alginate cultures induced IVD cell growth arrest whereas glucose deprivation was associated with increased proliferation. This enhanced cell proliferation was also evidenced by the formation of viable IVD cell clusters (Figure 3a, inset). Cytochemical positivity for SA-β-gal was significantly increased in serum-deprived alginate cultures (24% ± 3%) in comparison with all other conditions (Figure 3b). However, there was no evidence of serum-deprived cells in alginate becoming stellate, with all cells appearing spherical or ovoid. There was a significant decrease in cell viability in serum-deprived alginate cultures, in which only 63% ± 6% of cells remained alive at day 7 (see Additional file 1). Cell viability remained at approximately 95% in all other conditions (that is, in control or glucose- or oxygen-deprived cultures).

### Collagen production

**Figure 4:**
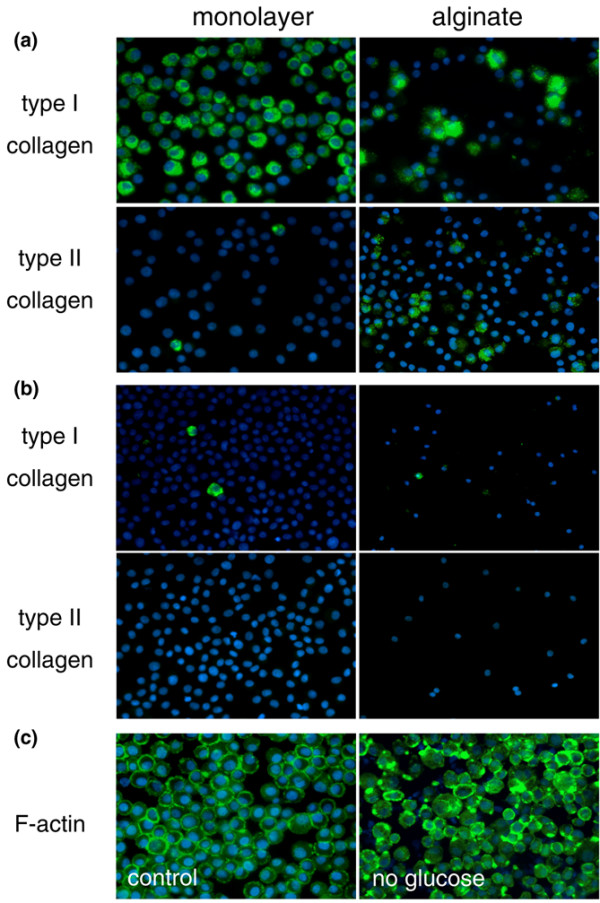
Collagen production by intervertebral disc (IVD) cells in monolayer and alginate cultures. Immunocytochemistry of cytospins was used to examine collagen production at day 7; representative images are shown. **(a) **In monolayer control cultures (left panels), collagen type I-immunopositive IVD cells were markedly more prevalent than collagen type II-immunopositive cells, indicative of a dedifferentiated phenotype [22]. This pattern of immunopositivity was reversed in alginate control cultures (right panels), suggesting that the cells in alginate had redifferentiated toward a chondrocytic phenotype [22]. **(b) **Immunopositivity for both types of collagen was markedly decreased in glucose-deprived cultures, both in monolayer (left panels) and alginate (right panels) culture. **(c) **Phalloidin-fluorescein isothiocyanate staining of IVD cells following monolayer (left panel) and alginate (right panel) culture in conditions of glucose-deprivation at day 7, demonstrating that the presence of filamentous actin (F-actin) was not markedly different in either condition (original magnification ×200).

Collagen type I immunopositivity in IVD cells was more prevalent in control monolayer cultures compared with control alginate cultures. Conversely, collagen type II was largely absent in monolayered IVD cells but was more prevalent in cells in alginate (Figure 4a). Serum or oxygen deprivation appeared to have little effect on these patterns of immunopositivity (data not shown). However, glucose deprivation was associated with a drastic reduction in the immunopositivity of both collagen type I and II, whether in monolayer or alginate cultures (Figure 4b). Staining for F-actin was used as an indicator of protein production. Hence, F-actin positivity was seen to similar extents in control and glucose-deprived IVD cells (Figure 4c).

The predominant phenotypic changes seen in IVD cells when cultured in the presence or absence of serum or glucose, both in monolayer and alginate culture systems, are summarised in Table 1. The minimal influence of oxygen levels on IVD cell growth and behaviour is omitted from this summary.

**Table 1:** The major phenotypes of bovine intervertebral disc cells in response to altered culture conditions

	Serum		Glucose	
Monolayer	With	Without	With	Without
Cell proliferation	↑↑↑	↓↓	↔	↑↑↑↑
Cell senescence	↔	↑↑↑	↔	↔
Cell death	↔	↔	↔	↔
Collagen synthesis	↑↑↑ Type I ↓ Type II	↔ Type I ↔ Type II	↔ Type I ↔ Type II	↓↓↓ Type I ↓ Type II

	Serum		Glucose	
Alginate	With	Without	With	Without

Cell proliferation	↓↓	↓↓↓	↔	↑
Cell senescence	↔	↑↑	↔	↔
Cell death	↔	↓↓	↔	↔
Collagen synthesis	↓ Type I ↑↑ Type II	↔ Type I ↔ Type II	↔ Type I ↔ Type II	↔ Type I ↓↓ Type II

↑, increased; ↓, decreased; ↔, no change (from control conditions).

## Discussion

Pathological conditions of the human IVD are associated with changes in IVD cell behaviour, including increased cell death [2,3], cell division [4], the appearance of stellate cells [5], and cell senescence [6-8]. What leads to these phenotypes is currently unclear; however, it is generally thought that nutritional deprivation may limit the capacity of IVD cells to function. Here, we have demonstrated that depriving IVD cells of serum, glucose, or oxygen has a variable influence on their growth and survival. Serum supplementation was required for continued IVD cell proliferation in monolayer cultures but was insufficient to drive proliferation to the same extent (as delineated by the Ki-67 index) in alginate cultures. Conversely, serum deprivation of monolayer cultures had no significant effect on cell viability but was associated with increased cell death in alginate cultures. In both culture systems, serum deprivation led to IVD cell growth arrest. These findings fit the general observation of various cell types that serum withdrawal induces cell growth arrest and/or apoptosis [27,28] whereas cell adhesion promotes cell survival [29]. We also found that serum deprivation was associated with increased SA-β-gal positivity in both culture systems and with the appearance of stellate cells in monolayer cultures. Both of these phenotypes have been linked with cell senescence or cellular adaptations to extracellular stresses [26,30]. As SA-β-gal staining was seen in cell populations that had undergone growth arrest, it can be concluded that this may be indicative of premature cell senescence. It remains to be determined how these findings relate to pathological human discs, where SA-β-gal-positive cells were seen most frequently in cell clusters [6] and have been associated with an increased catabolic activity [8]. In addition, Gruber and colleagues [31] have demonstrated increased levels of apoptosis in cells isolated from pathological human AF tissue in response to serum deprivation over a 10-day culture period of monolayer culture (from 0.1% to approximately 1%); this induction of cell death was inhibited by insulin-like growth factor-1 or platelet-derived growth factor. Zhao and colleagues [32] recently found that serum deprivation also induces apoptosis in rat AF cells, an effect that was exacerbated by interleukin-1-β. Hence, it is possible that the initial growth arrest and senescence that we have documented in response to serum deprivation may be followed by apoptotic cell death. Alternatively, NP cells and AF cells may require serum-derived survival factors to different extents. These areas warrant further investigation, as do the actions of individual growth and survival factors that may enter the disc from the vascular supply or that may be synthesised by IVD cells themselves [33].

The availability of oxygen and glucose to IVD cells is dependent on their transport from blood vessels and therefore on the degree of disc vascularisation (reviewed in [9,10]). As this varies according to age and pathology [17-21], the influence of oxygen and glucose on IVD cell growth may also be expected to vary. However, depriving IVD cells of oxygen alone had no marked effect on their growth or survival *in vitro*, either in monolayer or alginate culture. Risbud and colleagues [34] have shown that rat, sheep, and human IVD cells express hypoxia inducible factor-1-alpha (HIF-1-α), a transcription factor that is responsive to oxygen availability, even in normoxia. HIF-1-α expression was also only minimally induced and activated following hypoxia in culture. Furthermore, the same authors demonstrated that NP cells in rat discs, which are notochordally derived, do not appear to rely upon oxygen for their metabolism *in vivo *[35]. Although the central regions of the human IVD are more hypoxic than its periphery [36] (which may not be the case in smaller rat discs), one interpretation of the studies of Risbud and colleagues, along with our present findings, is that NP cells are adapted not to respond to hypoxia as readily as other cell types.

Depriving IVD cells of glucose resulted in increased cell proliferation, so long as serum (and to a lesser extent oxygen) was present, but a clear decrease in collagen production. The reasons for these responses are currently unclear; however, a similar relationship between glucose levels and proliferation was reported recently in Chinese hamster ovary (CHO) cells, which expanded in a low-glucose environment (6 mM) at a rate 1.7 times that seen in high-glucose conditions (33 mM) [37]. Similarly, exposure to higher levels of glucose (greater than 15 mM) was shown to inhibit cell proliferation in fibroblasts [38]. In our experiments, the proliferative response to glucose deprivation was dependent on the presence of serum, which in itself will contain glucose. As normal blood glucose levels vary between 4 and 8 mM (equating to 70 to 150 mg/dL), it may be assumed that the glucose levels in culture medium supplemented with 20% FCS will contain approximately 14 to 30 mg/dL. Hence, the precise relationships between levels of glucose and of other serum-derived factors in regulating IVD cell proliferation and collagen synthesis remain to be fully elucidated. It is noteworthy, however, that the increased cell proliferation seen in glucose-deprived alginate cultures resulted in the formation of NP cell clusters, which also form through increased cell proliferation in the NP *in vivo *and have been suggested to form part of an ineffective response to tissue damage [4].

## Conclusion

This study demonstrates that factors present in serum interact with other nutrients, notably glucose, to play a major role in regulating the behaviour of IVD cells. Furthermore, by manipulating the nutrient and serum supply to IVD cells *in vitro*, we have observed a number of cellular phenotypes akin to those seen in pathological human IVDs [2-7]. Hence, this study supports the conclusion that many of the cellular changes associated with IVD degeneration are likely to arise through the cells' response to altered vascularisation and nutrient supply.

## Abbreviations

AF = anulus fibrosus; DMEM = Dulbecco's modified Eagle's medium; F-actin = filamentous actin; FCS = foetal calf serum; FITC = fluorescein isothiocyanate; HIF-1-α = hypoxia inducible factor-1-alpha; IVD = intervertebral disc; NP = nucleus pulposus; SA-β-gal = senescence-associated beta-galactosidase.

## Competing interests

The authors declare that they have no competing interests.

## Authors' contributions

WEBJ helped to perform the experimental work, helped to conceive of the study, participated in its design and coordination, helped to perform the statistical analysis, and helped to draft the manuscript. SS helped to perform the experimental work and the statistical analysis and helped to draft the manuscript. SR helped to conceive of the study, participated in its design and coordination, and helped to draft the manuscript. All authors read and approved the final manuscript.

## Supplementary Material

Additional file 1 'Live/dead' staining of intervertebral disc (IVD) cells in alginate cultures. A representative image is shown of IVD cells in alginate cultured in serum-deprived conditions for 7 days; the pycnotic nucleus of a nonviable cell appears bright red, with viable cells appearing green (original magnification ×400). Click here for file
