# Supplementary figures and images for: The influence of serum, glucose and oxygen on intervertebral disc cell growth in vitro: implications for degenerative disc disease
Source: Arthritis Res Ther. 2008 Apr 23;10(2):R46. doi: 10.1186/ar2405 (PMC2453766; doi:10.1186/ar2405)

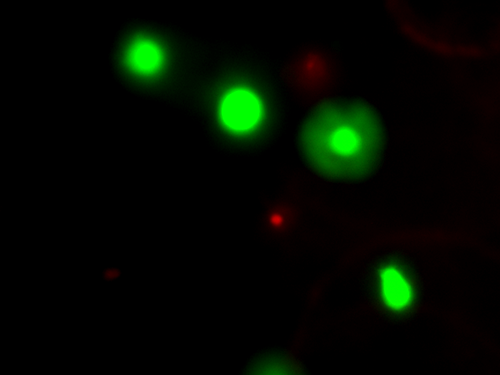

Supplement: Additional file 1 — 'Live/dead' staining of intervertebral disc (IVD) cells in alginate cultures. A representative image is shown of IVD cells in alginate cultured in serum-deprived conditions for 7 days; the pycnotic nucleus of a nonviable cell appears bright red, with viable cells appearing green (original magnification ×400). [file ar2405-S1.tiff]
